# A Survey of Attitudes towards the Clinical Application of Systemic Inflammation Based Prognostic Scores in Cancer

**DOI:** 10.1155/2015/842070

**Published:** 2015-10-04

**Authors:** David G. Watt, Campbell S. Roxburgh, Mark White, Juen Zhik Chan, Paul G. Horgan, Donald C. McMillan

**Affiliations:** ^1^Academic Unit of Surgery, School of Medicine, University of Glasgow, Glasgow Royal Infirmary, Glasgow G31 2ER, UK; ^2^University of Glasgow, Glasgow Medical School, University Avenue, Glasgow G12 8QQ, UK

## Abstract

*Introduction.* The systemic inflammatory response (SIR) plays a key role in determining nutritional status and survival of patients with cancer. A number of objective scoring systems have been shown to have prognostic value; however, their application in routine clinical practice is not clear. The aim of the present survey was to examine the range of opinions internationally on the routine use of these scoring systems.* Methods.* An online survey was distributed to a target group consisting of individuals worldwide who have reported an interest in systemic inflammation in patients with cancer.* Results.* Of those invited by the survey (*n* = 238), 65% routinely measured the SIR, mainly for research and prognostication purposes and clinically for allocation of adjuvant therapy or palliative chemotherapy. 40% reported that they currently used the Glasgow Prognostic Score/modified Glasgow Prognostic Score (GPS/mGPS) and 81% reported that a measure of systemic inflammation should be incorporated into clinical guidelines, such as the definition of cachexia.* Conclusions.* The majority of respondents routinely measured the SIR in patients with cancer, mainly using the GPS/mGPS for research and prognostication purposes. The majority reported that a measure of the SIR should be adopted into clinical guidelines.

## 1. Introduction

Cancer remains a major problem worldwide with 12.7 million new cases diagnosed in 2008. In the UK alone, 331,000 people were diagnosed with cancer in 2011 [1]. Despite major advances in detection and treatment of cancer as well as the introduction of several cancer screening programmes, outcomes following cancer remain poor with only half of people diagnosed with cancer surviving at 5 years [1].

Allocation of patients to the correct form of treatment, be that surgical, oncological, or palliative, remains a difficult decision. However, if patients were allocated to the most appropriate treatment, then outcomes for all patients would improve, irrespective of new, more effective treatments. Traditionally, in those with early stage operable disease the treatment decision has been made largely based on staging of the cancer itself for example the Tumour, Node, Metastasis (TNM) staging system whereas in advanced stage inoperable disease the treatment decision has been made largely based on the general health and fitness and whether the patient had lost weight (cachexia).

In the last decade or so it has become apparent that a host inflammatory response, in particular the systemic inflammatory response, plays a key role in determining cachexia and the survival of patients with cancer [2, 3]. With this new knowledge, a number of prognostic scoring systems that provide an objective measurement of the systemic inflammatory response have been developed and have been shown to have prognostic value in patients with cancer. These include the Glasgow Prognostic Score/modified Glasgow Prognostic Score (GPS/mGPS), neutrophil-lymphocyte ratio (NLR), platelet-lymphocyte ratio (PLR), white cell-lymphocyte ratio (WLR), and others [4, 5]. The mGPS (combination of the values of preoperative serum albumin and C-reactive protein) and the NLR (ratio of neutrophil and lymphocyte counts) are the most widely reported prognostic scores worldwide and both have been shown to have prognostic value in a variety of common solid tumours [6–8]. For example, by the end of 2012, the GPS/mGPS had been shown to have independent prognostic value in cancer patients in 51 studies involving 28,500 patients [6]. Furthermore, the NLR has been shown to have independent prognostic value in 100 studies involving greater than 40 000 patients, with greater than 50% of these studies published since the start of 2012 [9].

Despite the plethora of reported studies for these prognostic scores, their value in routine clinical practice either as tools to stratify patients in terms of outcomes or for consideration for therapies such as adjuvant chemotherapy or in clinical trials is not clear. With this in mind, the aim of the present survey, in an international cohort, was to examine the range of opinions on the routine use of systemic inflammation based prognostic scoring systems and their potential incorporation into clinical guidelines.

## 2. Methods

A worldwide survey designed to establish opinions on the use of systemic inflammation based prognostic scoring systems was created. This was a web-based survey that included 10 questions on “systemic inflammation based prognostic scores in cancer” as follows.

*Survey Questions*

1. What is your discipline (surgeon/oncologist/pathologist, etc.) and in which country are you based?
2. Do you or your colleagues routinely assess the systemic inflammatory response as part of the clinical assessment of patients with cancer?
   Since 2008, could you estimate how many patients have been assessed (a) in total and (b) per year?
3. If you answered yes to question (2), for what purpose?
   Audit
   Prognostication
   Treatment Allocation
   Research
4. If you answered yes to question (2), what measure of the systemic inflammatory response do you use?
   GPS
   NLR
   Other
5. Would you use a measure of the systemic inflammatory response to stratify patients entering into clinical trials?
6. If you answered yes to question (5), which would you prefer to use?
   GPS
   NLR
   Other
7. In which clinical scenario do you think a measure of the systemic inflammatory response offers most benefit to patients?
   In making decisions about allocation of surgical treatment for primary operable disease
   In making decisions on allocation of neoadjuvant treatment
   In making decisions on allocation of adjuvant treatment
   In making decisions on palliative chemotherapy
8. Do you think that a measure of the systemic inflammatory response should be adopted into clinical guidelines?
9. If yes, which would you prefer to use?
   GPS
   NLR
   Other
10. If you do not think that a measure of the systemic inflammatory response is useful in the routine clinical assessment of cancer patients, please comment.

The survey was generated through the SurveyMonkey website (http://www.surveymonkey.com/, SurveyMonkey, Paulo Alto, USA) and the access link emailed to the target group. The target group was selected primarily from two recent reviews [6, 7] and by performing a more recent literature search for articles using the keywords cancer, inflammation, recurrence, survival, mGPS, and NLR. This literature search was performed at the end of January 2014. Once a comprehensive list of articles was obtained, the email addresses of corresponding authors from each article formed the basis of a mailing list for distribution. The email sent out clearly stated that the aim of the survey was to establish whether there was a role for the application of systemic inflammation based prognostic scores in routine clinical practice and research and that participation was voluntary. Software on the website ensured duplication of responses from the same individual was not recorded. No incentives were used to promote or encourage participation.

The survey was first sent out on 26th February 2014 with a reminder sent out one week later. The survey remained open for 4 weeks and was closed on the 26th March 2014. Data was analysed and graphs of results were compiled using Microsoft Excel 2007 (Redmond, WA, USA).

## 3. Results

**Figure 1:**
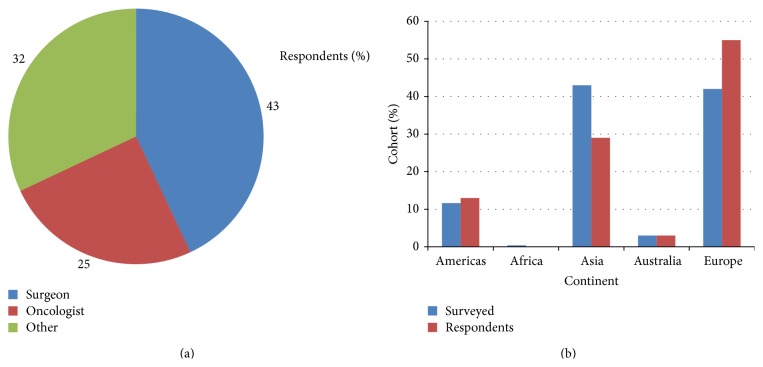
(a) What is your discipline? (Respondents = 60) and (b) in which country are you based? (Respondents = 31).

In February 2014, the survey was emailed to 238 individuals worldwide who had published articles related to systemic inflammation in patients with cancer. 43% were from Asia, 42% from Europe, 12% from America, and 3% from Australia. The response to survey question (1) is shown in Figures 1(a) and 1(b). In total, 60 people completed the survey (25%). 26 respondents (43%) were surgeons, 15 (25%) oncologists, and 19 (32%) from other medical specialties. The proportion of respondents is shown in Figure 1(b) with 55% of respondents being from Europe, 29% from Asia, 13% from Americas, and 3% from Australia.

In response to question (2), 39 (65%) of the respondents answered yes that they routinely measured the systemic inflammatory response in patients with cancer. The median number of patients each participant assessed per year was 100 and the median number of patients each participant assessed in total was 330.

**Figure 2:**
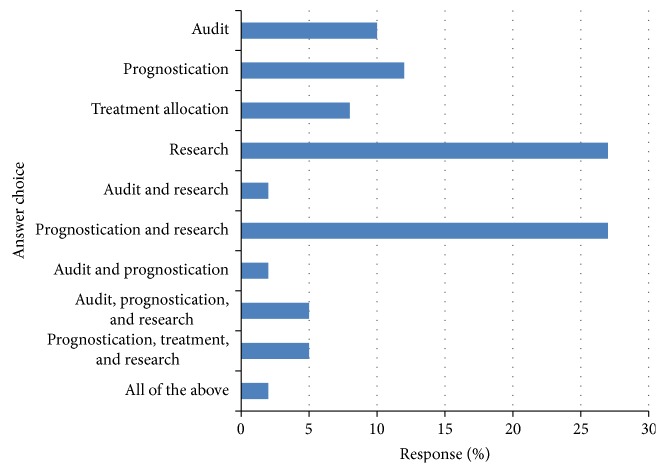
For what purpose do you measure the systemic inflammatory response? Respondents (*n* = 41).

The response to question (3) is shown in Figure 2. Of the respondents, 11 (27%) reported its use for the purpose of prognostication and research, 11 (27%) reported its use for research purposes alone, 5 (12%) reported its use for the purpose of prognostication alone, 4 (10%) reported its use for audit purposes, and 3 (8%) reported its use for the purpose of treatment allocation.

**Figure 3:**
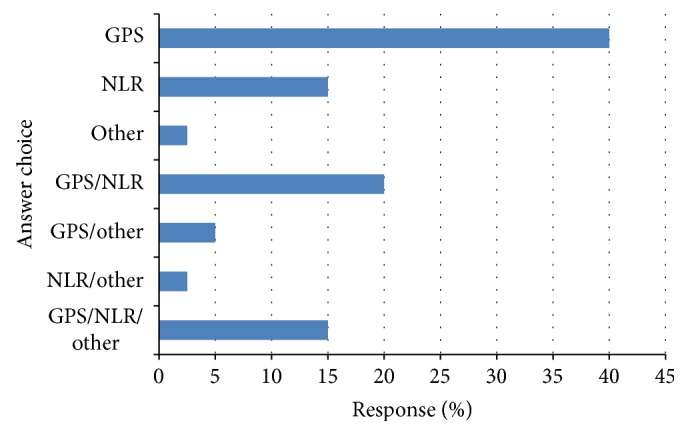
What measure of the systemic inflammatory response do you use? Respondents (*n* = 40).

The response to question (4) is shown in Figure 3. Of those who responded, 16 (40%) answered that the measure of the systemic inflammatory response they used was the GPS, 8 (20%) the GPS/NLR, and 6 (15%) the NLR alone.

**Figure 4:**
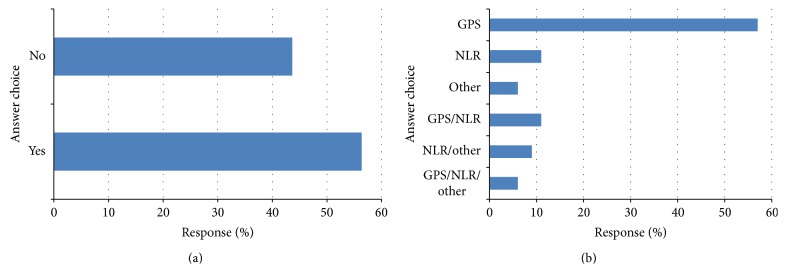
(a) Would you use a measure of the systemic inflammatory response to stratify patients entering into clinical trials? Respondents (*n* = 55) and (b) which measure of the systemic inflammatory response would you use to stratify patients entering into clinical trials? Respondents (*n* = 35).

The response to question (5) is shown in Figure 4(a). Of the respondents, 31 (56%) answered yes they would use a measure of the systemic inflammatory response to stratify patients entering clinical trials.

The response to question (6) is shown in Figure 4(b). Of the respondents, 20 (57%) answered that they would use the GPS, 4 (11%) the NLR, and 4 (11%) the GPS/NLR for stratifying patients entering clinical trials.

**Figure 5:**
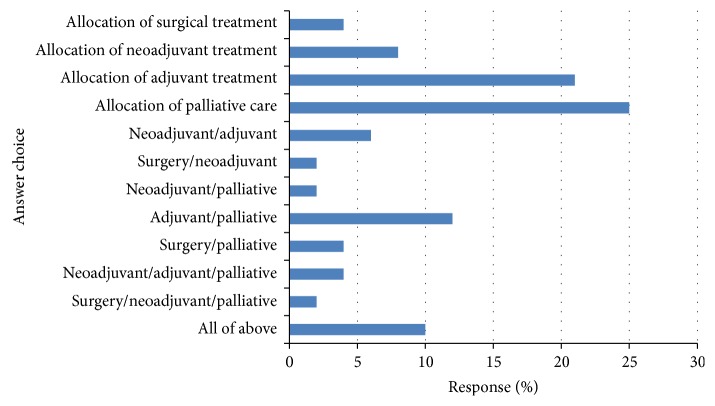
In which clinical scenario do you think a measure of the systemic inflammatory response offers most benefit to patients? Respondents (*n* = 49).

The response to question (7) is shown in Figure 5. Of the respondents, 12 (25%) reported that the clinical scenarios where a measure of the systemic inflammatory response offers most benefit were making decisions on palliative chemotherapy, 10 (21%) making decisions on allocation of adjuvant therapy, 6 (12%) making decisions about either adjuvant therapy or palliative chemotherapy, and 5 (10%) all 4 categories. Only 2 (4%) reported on making decisions on allocation of surgical treatment.

**Figure 6:**
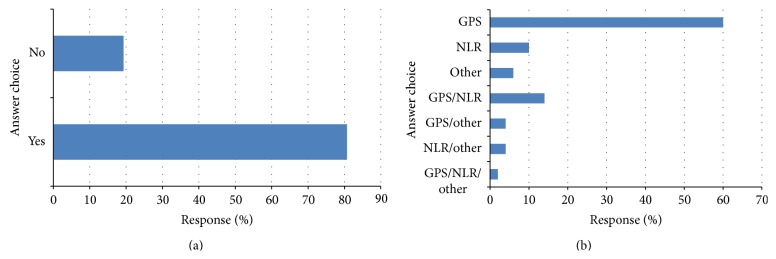
(a) Do you think that a measure of the systemic inflammatory response should be adopted into clinical guidelines? Respondents (*n* = 57) and (b) which measure of the systemic inflammatory response respondants think should be included in clinical guidelines? Respondents (*n* = 50).

The response to question (8) is shown in Figure 6(a). Of the respondents, 46 (81%) answered yes to whether a measure of the systemic inflammatory response should be adopted into clinical guidelines.

The response to question (9) is shown in Figure 6(b). Of those who responded, 30 (60%) answered that the measure of the systemic inflammatory response they would prefer to use in clinical guidelines was the GPS, 7 (14%) GPS/NLR, and 5 (10%) NLR.

## 4. Discussion

The results of the present study showed that the majority of respondents routinely measured the systemic inflammatory response and used the GPS/mGPS, mainly for research and prognostication purposes and that the majority of respondents reported that a measure of the systemic inflammatory response should be adopted into clinical guidelines.

A small number of people responded to our survey (25%) although this rate falls within the average response rate of between 20 and 30% [10]. Factors that are known to improve the survey response rate include incentives, reduced survey length, reduced complexity of questions, and reminder emails [10]. In the present study the questions were intentionally simple and limited to 10 in total and a reminder email was sent to encourage respondents but did not employ any incentive for completing the survey.

The survey was sent to potential participants worldwide with the majority to Asia and Europe. The majority of respondents of this survey were surgeons (43%) with oncologists making up a quarter of respondents. The location of the respondents did not closely match the locations of the potential survey participants. Those invited to participate were mainly from Asia and Europe; however, only 29% of respondents were from Asia while 55% were from Europe. Perhaps this lack of response from Asia is due to cultural differences which were not present in those from Europe or due to greater language barriers. Whatever the reason, the poor response rate from Asia was disappointing given that the majority of work using these prognostic scores has been carried out in Europe and Asia. In the present study, respondents were asked to estimate how many patients with cancer they had assessed using these systemic inflammation based scores in each year. The response was approximately 100 per year. With this volume of work it could be considered that those who responded were specialists and had an interest in systemic inflammation based scores.

It has been widely reported that markers of the systemic inflammatory response are good prognostic markers in patients with cancer. The majority of survey respondents reported that they routinely assessed the systemic inflammatory response in patients with cancer and the majority used this assessment for research or prognostication purposes. This is not unexpected since the majority of studies examining these scoring systems were performed for research purposes or were performed retrospectively to aid prognostication of patients into high and low risk groups. Whilst CRP has been shown to have prognostic value in a number of tumours, the mGPS, which utilises a combination of CRP and albumin at standard thresholds, has been shown to have superior prognostic value and obviates the problem of different CRP threshold values being used within and across different tumour types. In the present study, the majority of respondents reported that they would use GPS/mGPS as their method of assessing the systemic inflammatory response. This would appear to be consistent with the literature and whilst the participants of this survey have an interest in this field, it was not clear, prior to this survey, what views they had on the clinical application of systemic inflammation based prognostic scores, in particular which, if any, score that they would prefer to use clinically.

Interestingly, only a small number of respondents reported that they used assessment of the systemic inflammatory response to determine treatment allocation and this is an area where proponents of these scoring systems would hope to expand their use in order to better stratify patients to appropriate treatment modalities [11]. Of the survey respondents, 56% reported that they would use a measure of the systemic inflammatory response to stratify patients entering into clinical trials and 57% said they would choose mGPS/GPS for this. Moreover, of the survey respondents, 25% reported that these scores were used in making decisions about palliative chemotherapy, 21% in making decisions about allocation of adjuvant therapy, and 12% in making decisions either about adjuvant therapy or palliative chemotherapy. Only 4% reported that a measure of the systemic inflammatory response would be of benefit in making decisions about allocation of surgical treatment. This is of interest as the majority of respondents were surgeons, with the majority of research in these scoring systems having been undertaken by surgeons, yet the consensus was that it would not be of benefit to allocate surgical treatment based on these scoring systems. The basis of this approach is not clear. However it may be that surgeons wish to operate on all patients with potentially curable disease. It remains to be seen whether this approach will be maintained in the long term, particularly in aggressive cancers such as pancreatic cancer where neoadjuvant therapy is increasingly used as first line therapy.

Furthermore, recent work has suggested that markers of the systemic inflammatory response may be useful as a therapeutic target. The recent addition of an antiangiogenic monoclonal antibody to VEGF therapy, such as Bevacizumab to standard chemotherapy regimens, has resulted in improved efficacy of these regimens. However, recent studies have reported that patients with a raised neutrophil count, high NLR or mGPS 1 or 2, received no significant survival benefit from these regimens [12–14]. In addition, Botta and colleagues reported in their study that preoperative systemic inflammatory status was a marker of resistance to bevacizumab therapy [13]. Also, recent work has suggested that the mGPS may be useful in stratifying oncological treatment. Hurwitz et al. recently reported that Ruxolitinib (a Janus Kinase 1 (JAK1)/Janus Kinase 2 (JAK2) inhibitor) along with capecitabine improved overall survival and progression free survival in patients with metastatic pancreatic cancer with inflammation characterised by mGPS 1 or 2 [15].

Of the survey respondents, 80% reported that they felt that a measure of the systemic inflammatory response should be adopted into clinical guidelines and 60% reported that GPS/mGPS would be their preference. For example, cancer cachexia affects greater than 50% of patients with advanced disease and its clinical definition and symptoms have been intensively discussed in recent years [16, 17]. Recently, the European School of Oncology Task Force conducted a review of the literature on cancer cachexia. They concluded that cachexia is a complex process but that, along with anorexia, the presence of a systemic inflammatory response results in the features of the disease [16]. Furthermore, Douglas and McMillan (2014) recently proposed that the mGPS be used as the basis for formation of an objective and clinically relevant definition of cachexia [17]. The findings of the present study would appear to confirm that the mGPS is the most commonly used systemic inflammation based score and therefore appropriate for forming the basis of an objective definition of cancer cachexia.

The present study has a number of possible limitations. Firstly, respondents did not have to enter their location in order to complete the questionnaire, meaning the location for all the respondents was not obtained. In all surveys there is a tension between making the sample size as large as possible in order to eliminate bias and asking questions appropriate to those surveyed. In the present survey, we targeted those with a known interest in systemic inflammation based prognostic scores (those who had already published in this field) in order to maximise the number of appropriate and meaningful responses. The mGPS and NLR are the most popular scores as they have the largest evidence base. Although other systemic inflammation based prognostic scores such as the derived NLR (dNLR), lymphocyte monocyte ratio (LMR), and platelet-lymphocyte ratio (PLR) have been reported, they have not established a sufficient body of evidence in the literature. Moreover, where they have been directly compared, the mGPS had the greatest prognostic value in patients with cancer, independent of age, sex, deprivation, and tumour stage [4, 18]. Therefore, it is likely that the results of this survey reflect the reality of attitudes towards the application of these scores in those individuals with an interest in the field. It was of interest that 43% of the respondents were surgeons. This may reflect the activity of surgeons in this field. Indeed, it is recognised that surgeons are key members of the multidisciplinary team (MDT) that decides treatment allocation. Irrespectively, this would confirm that the survey was directed at clinicians in routine clinical practice.

In summary, the present study has shown that, in those who responded, the majority routinely measured the systemic inflammatory response in patients with cancer, with the majority using the GPS/mGPS, mainly for research and prognostication purposes. The majority reported that these scoring systems were of most clinical benefit in making decisions on adjuvant therapy and palliative chemotherapy and that the systemic inflammatory response, as evidenced by the GPS/mGPS, should be adopted into clinical guidelines, such as a new, objective and clinically relevant definition of cancer cachexia.
